# TRIM26 inhibits clear cell renal cell carcinoma progression through destabilizing ETK and thus inactivation of AKT/mTOR signaling

**DOI:** 10.1186/s12967-024-05273-w

**Published:** 2024-05-21

**Authors:** Di Zheng, Jinzhuo Ning, Hao Deng, Yuan Ruan, Fan Cheng

**Affiliations:** Department of Urology, Renmin Hospital of Wuhan University, Wuhan, 430060 Hubei Province P. R. China

**Keywords:** TRIM26, ccRCC, mTOR signaling pathway, ETK, Ubiquitination

## Abstract

**Background:**

Tripartite motif-containing 26 (TRIM26), a member of the TRIM protein family, exerts dual function in several types of cancer. Nevertheless, the precise role of TRIM26 in clear cell renal cell carcinoma (ccRCC) has not been investigated.

**Methods:**

The expression of TRIM26 in ccRCC tissues and cell lines were examined through the use of public resources and experimental validation. The impacts of TRIM26 on cell proliferation, migration, invasion, and epithelial-mesenchymal transition (EMT) process were determined via CCK-8, colony formation, EdU incorporation, wound healing, Transwell invasion, Western blot, and Immunofluorescence assays. RNA-seq followed by bioinformatic analyses were used to identify the downstream pathway of TRIM26. The interaction between TRIM26 and ETK was assessed by co-immunoprecipitation, qRT-PCR, Western blot, cycloheximide (CHX) chase, and in vivo ubiquitination assays.

**Results:**

We have shown that TRIM26 exhibits a downregulation in both ccRCC tissues and cell lines. Furthermore, this decreased expression of TRIM26 is closely linked to unfavorable overall survival and diseases-free survival outcomes among ccRCC patients. Gain- and loss-of-function experiments demonstrated that increasing the expression of TRIM26 suppressed the proliferation, migration, invasion, and EMT process of ccRCC cells. Conversely, reducing the expression of TRIM26 had the opposite effects. RNA sequencing, coupled with bioinformatic analysis, revealed a significant enrichment of the mTOR signaling pathway in the control group compared to the group with TRIM26 overexpression. This finding was then confirmed by a western blot assay. Subsequent examination revealed that TRMI26 had a direct interaction with ETK, a non-receptor tyrosine kinase. This interaction facilitated the ubiquitination and degradation of ETK, resulting in the deactivation of the AKT/mTOR signaling pathway in ccRCC. ETK overexpression counteracted the inhibitory effects of TRIM26 overexpression on cell proliferation, migration, and invasion.

**Conclusion:**

Our results have shown a novel mechanism by which TRIM26 hinders the advancement of ccRCC by binding to and destabilizing ETK, thus leading to the deactivation of AKT/mTOR signaling. TRIM26 shows promise as both a therapeutic target and prognostic biomarker for ccRCC patients.

**Supplementary Information:**

The online version contains supplementary material available at 10.1186/s12967-024-05273-w.

## Introduction

Renal cell carcinoma (RCC), a very deadly disease of the urinary system, ranked as the ninth most common cancer in females and the sixth most common cancer in males. It was anticipated that there were over 400,000 new cases of RCC worldwide in 2020 [1, 2]. Clear cell renal cell carcinoma (ccRCC) is the overwhelming majority subtype of renal cell carcinoma (RCC), making up about 75% of all RCC cases. Papillary RCC (pRCC) and chromophobe RCC (chRCC) account for around 15–20% and 5% of RCC cases, respectively [3, 4]. ccRCC generally originates in the tubular epithelium of the kidney and is defined by the presence of cytoplasmic lipid deposits, which is the foundation of its pathological nomenclature [5]. ccRCC is characterized by a high degree of malignancy, often leading to early metastasis and recurrence. Previously, radical nephrectomy has been considered the most essential therapeutic approach for localized clear cell renal cell carcinoma (ccRCC) [6]. Nevertheless, around one-third of ccRCC patients diagnosed with localized illnesses have tumor recurrence or metastasis after undergoing surgical resection, with the lung, bone, and liver being the most frequently afflicted locations [7–10]. In general, around 25–30% of recently identified ccRCC cases exhibited either local invasion or distant metastasis. This is a significant obstacle in effectively treating these advanced ccRCC cases, and the prognosis for these patients is unfavorable, with a five-year overall survival rate of fewer than 15% [11, 12]. Despite the significant advancements in the treatment of metastatic cancer via the approval of immunotherapy and targeted therapy in recent decades, these tactics have not shown adequate results in the treatment of metastatic ccRCC [13, 14]. Hence, it was essential to uncover new molecular targets involved in the development and advancement of ccRCC in order to enhance the therapy of advanced cases.

Tripartite motif-containing 26 (TRIM26), a protein belonging to the TRIM family, has been shown to have significant involvement in several physiological and pathological processes, including ferroptosis, inflammation, antiviral immunity, and cancer [15–17]. Structurally, TRIM26 exhibited a high degree of conservation in its N-terminal region, which included three domains: the RING finger domain, the B-box motifs, and a coiled-coil region [18]. It is worth mentioning that proteins containing a RING finger domain often possess E3 ligase activity, which controls the process of ubiquitination of downstream substrates. Many members in the TRIM family, such as TRIM26, have E3 ubiquitin ligase activity [19]. TRIM26 has recently received significant interest due to its dual function in carcinogenesis and progression of different types of malignancies. TRIM26 exhibited significant upregulation in non-small cell lung cancer (NSCLC) and facilitated NSCLC cell proliferation and metastasis by inducing K48-linked polyubiquitination of PBX1 [20]. In glioblastoma, TRIM26 could stabilize SOX2 protein, a pluripotency transcription factor, by inhibiting the interaction of SOX2 with its targeting E3 ubiquitin ligase WWP2 to promote the tumorigenicity of glioblastoma stem cells [21]. In addition to its involvement in oncogenesis, TRIM26 may also function as a tumor suppressor. For example, TRIM26 exhibited decreased expression in hepatocellular carcinoma (HCC) and suppressed cell proliferation and migration. Conversely, USP39, a deubiquitinase, had a contrasting impact. Mechanistically, TRIM26 functioned as an antagonistic protein of USP39. TRIM26 and USP39 maintain the protein level of ZEB1 by controlling its ubiquitination, hence influencing the development of HCC [22]. Nevertheless, the specific function and potential mechanism by which TRIM26 involves in the progression of ccRCC have not yet been fully understood.

This work demonstrates that TRIM26 is downregulated in ccRCC, as determined by the use of bioinformatic methods and experimental validation. Furthermore, the study reveals that lower expression of TRIM26 is associated with worse overall survival and disease-free survival in ccRCC patients. Gain- and loss-of-function tests demonstrated that the overexpression of TRIM26 suppressed cell proliferation, migration, invasion, and the epithelial-mesenchymal transition (EMT) process in clear cell renal cell carcinoma (ccRCC). Conversely, the downregulation of TRIM26 had the opposite effects. TRIM26 may directly interact with ETK and enhance its ubiquitination, hence deactivating the AKT/mTOR signaling pathway in ccRCC and impeding the development of ccRCC. Our investigation revealed valuable information about the tumor suppressive function of TRIM26 in ccRCC and its ability to predict outcomes. TRIM26 shows promise as both a possible therapeutic target and a predictive biomarker in ccRCC.

## Materials and methods

### Bioinformatic analysis

The expression data of ccRCC tissues and adjacent normal tissues were downloaded from TCGA (https://portal.gdc.cancer.gov/) and GEO (https://www.ncbi.nlm.nih.gov/geo/) databases, and were further utilized to compare TRIM26 expression between ccRCC tissues and normal tissues. The detailed information of the three GEO datasets (GSE53757, GSE40435, and GSE46699) were listed in Table 1. Comparison of the difference in overall survival and diseases-free survival between TRIM26^high^ and TRIM26^low^ groups were conducted using the online GEPIA database (http://gepia.cancer-pku.cn/detail.php).

**Table 1:** The detailed information of the three GEO datasets

GEO	Platform	Sample	Normal	Tumor	Year	Reference
GSE53757	GPL570	ccRCC	72	72	2014	Von Roemeling CA et al. [23]
GSE40435	GPL10558	ccRCC	101	101	2013	Wozniak M et al. [24]
GSE46699	GPL570	ccRCC	63	67	2014	Serie DJ *et a*l [25]

### Tissue collection

All primary ccRCC tissues and matched normal tissues were collected from the Department of Urology, Renmin hospital of Wuhan university between July 2022 to January 2023, with the approval by the Research Ethics Committee of Renmin Hospital of Wuhan University. The histological characteristic of the ccRCC sample were identified by two independent pathologists. After being resected, tissue samples were fixed with 4% formaldehyde, or frozen in lipid nitrogen immediately and then stored in -80 °C until being utilized in the subsequent experiments.

### Cell culture

Human ccRCC cell lines (786-O, ACHN, and 769-P), normal renal cells HK-2, and human embryonic kidney cells HEK 293T cell lines were obtained from the Cell Bank of Type Culture Collection (CBTCC, Chinese Academy of Sciences, Shanghai, China). The Caki-1 and OS-RC2 cells were purchased from the Procell Life Science and Technology (Wuhan, China). The DMEM medium (Hyclone, USA) was applied for ACHN and HEK 293T cells culturing, and the RPMI 1640 medium (Hyclone, USA) was used for 786-O and 769P cells culturing. OS-RC2 and HK-2 cells were maintained in α-MEM medium and Caki-1 cells were cultured in McCoy’s 5a medium. All the medium were supplemented with 10% fetal bovine serum (FBS; Gibco, USA) and 1% antibiotics (Servicebio Technology, Wuhan, China). Cells were cultured in a humidified chamber at 37 °C and an atmosphere of 5% CO2.

### Lentiviral infection and transient transfection

Lentiviral vector for ectopic expression of full-length TRIM26, and lentiviral shRNA expression vectors for human TRIM26, their corresponding control vectors were purchased from OBiO Technology (Shanghai, China). For lentiviral infection, ccRCC cells were seeded into 6-well plate and cultured until cell density reaching approximately 50–60%. After being washed with PBS and replacing with fresh medium, 786-O and Caki-1 cells were infected by lentivirus according to the manufacturer’s instruction. The antibiotic-resistant infected cells were selected in puromycin (5 µg/mL) for 2 weeks to achieve stable TRIM26 overexpression or knockdown. The overexpression plasmids were constructed by cloning full length CDS region of TRIM26 or ETK into FLAG-pcDNA3.1 or HA-pcDNA3.1 vector. SiRNA specific targeting ETK was purchased from Sangon Biotech (Shanghai, China). For transient transfection, 3 µg plasmid or 4 µl siRNA were mixed with 200 µl Opti-MEM culture medium containing 5 µl HighGene transfection reagent (ABclonal Technology, China). After incubation for 10 min at 4 °C, the mixture was added into each well of the 6-well plate. 48 h after transfection, cells were harvest and were utilized for the subsequent biological and biochemical experiments. qRT-PCR and western blot experiments were carried out to evaluate the overexpression or knockdown efficiencies.

### CCK-8, colony formation assay

786-O and Caki-1 cells were digested through trypsinization and prepared to be single-cell suspension in culture medium. For CCK-8 assay, cells were seeded into 96-well plates at a density of 3 × 10^3^ per well and five wells each group (*n* = 5). At the prescribed time point (0, 24, 48, 72 h), 10 µl CCK-8 reagents (Dojindo, Tokyo, Japan) were added into each well. After incubation for 1 h at 37 °C in dark, the optical density (OD) value was measured using a microplate reader. For colony formation assay, cells were seeded into 6-well plates in a number of 1 × 10^3^ per well, and cultured for two weeks. After washing three times with PBS, cells were fixed with 4% paraformaldehyde and then stained with 1% crystal violet to visualized colonies. The number of visible colonies was determined by ImageJ software.

### EdU incorporation assay

EdU incorporation assay was conducted using an EdU Apollo DNA in vitro kit (RiboBio, Guangzhou, China) according to the manufacturer’s protocol. Briefly, cells were seeded into 24-well plates and incubated with EdU solution (10 mM) for 2 h. After being washed three times with PBS, the membrane was permeabilized with 0.5% TritonX-100 and the nuclei was then stained with DAPI (1 µg/ml) for 20 min at room temperature in dark. The original images were photographed in five random fields using a fluorescence microscopy, and the proportion of EdU positive cells were calculated using ImageJ software.

### Wound healing assay

Cells were seeded into 6-well plates to form a confluent monolayer. Then, the monolayer was scratched using a conventional sterile pipette tip to create a vertical wound. After being washed once with PBS, cells were cultured in serum free medium for another 24 h. Cells were observed and photographed at 0 and 24 h using an inverted microscopy. The wound healing rate was determined by calculating the area of wound using ImageJ software.

### Transwell invasion assay

Briefly, 5 × 10^3^ cells in 200 µl serum free medium were added into the upper chamber of the Matrigel-coated transwell insert (Coring, USA). The lower chamber was supplemented with 600 µl medium containing 20% FBS. After incubation for 36 h, cells on the upper surface of the membrane were moved out carefully, and cell invaded through the membrane to the lower surface were fixed with 4% paraformaldehyde and then stained with 1% crystal violet. Images were photographed in five random fields using an inverted microscopy and the number of invaded cells was calculated by averaging the counts of the five fields.

### RNA isolation, qRT-PCR, and RNA-Seq

Total RNA was isolated from tissues and cells using TRIzol reagent (Thermo Fisher Scientific, USA). After measuring the concentration of total RNA, 1 µg RNA was utilized to be reversely transcribed into cDNA using ReverTrace qRT-PCR Kit (Toyobo, China). qRT-PCR was carried out using 2× SYBR Green mix (Bio-Rad, USA) under standard condition, and each reaction of qRT-PCR was performed with 10 µl SYBR Green mix, 3 µl primers, 2 µl cDNA, and 5 µl ddH_2_O. Relative gene expression was calculated by the 2^−ΔΔCt^ method and *GAPDH* expression was regarded as internal control. The sequences of the primers were listed as follow: *TRIM26*, forward: 5′-GTGACCTGCTCCATCTGTCT-3′ and reverse: 5′-CGCTCAATGTTCTCCACCAG-3′. *ETK*, forward: 5′-CGATGTCTGTGAAGGCATGG-3′ and reverse: 5′-CCATACGTCTGACTTGCTGC-3′. *GAPDH*, forward: 5′-CCATCTTCCAGGAGCGAGAT-3′ and reverse: 5′-TGCTGATGATCTTGAGGCTG-3′.

### Western blot

Total protein was extracted from tissues and cells using RIPA lysis buffer (CST, USA) supplemented with 1% protease and phosphatase inhibitor (MCE, USA). After determining the concentration of the total protein according to the BCA kit (Solarbio Science & Technology Co. Ltd, Beijing, China), total protein was diluted into 2 µg/µl using RIPA lysis buffer and 5×SDS loading buffer (Servicebio Technology, Wuhan, China). Equal amounts of proteins (20 µg) in each group were utilized for SDS-PAGE separation, followed by electro-transfer onto a PVDF membrane (Merck Millipore, USA). After blocking with skim milk for 1 h at room temperature, the membrane was incubated with primary antibodies at 4 °C overnight. The next day, the membrane was washed three times with TBST and then incubated with horseradish peroxidase-conjugated secondary antibodies (Servicebio Technology, Wuhan, China). The immunoblotting was visualized using the enhanced chemiluminescence reagent (Beyotime, Shanghai, China). Relative protein level was calculated using ImageJ software and GAPDH was served as a loading control. The information for the primary antibodies was listed as follow: TRIM26 (Cat: sc-393,832, Santa Cruz Biotechnology), ETK (Cat: sc-376,686, Santa Cruz Biotechnology), GAPDH (Cat: GB15004-100, Servicebio Technology), AKT (Cat: #4691, Cell Signaling Technology), p-AKT (Cat: #4060, Cell Signaling Technology), mTOR (Cat: #2983, Cell Signaling Technology), p-mTOR (Cat: #5536, Cell Signaling Technology), 4EBP1 (Cat: #9644, Cell Signaling Technology), p-4EBP1 (Cat: #2855, Cell Signaling Technology), E-cadherin (Cat: #14,472, Cell Signaling Technology), N-cadherin (Cat: GB111273-100, Servicebio Technology), Vimentin (Cat: GB111308-100, Servicebio Technology), Ub (Cat: #3936, Cell Signaling Technology), FLAG (Cat: #14,793, Cell Signaling Technology), HA (Cat: #3724, Cell Signaling Technology).

### Co-immunoprecipitation (Co-IP)

Co-immunoprecipitation assay was conducted using the Classic Magnetic Protein A/G IP/Co-IP Kit (Epizyme Biotech, Shanghai, China). Briefly, cells were harvested and were then lysed using IP lysis buffer containing 1% protease inhibitor. After incubation for 30 min at 4 °C with rotation, the cell lysates were centrifuged at 12,000×g at 4 °C for 10 min. Subsequently, the supernatants were transferred into another sterile tubes, and were immunoprecipitated with specific antibodies or IgG overnight at 4 °C with constant gentle shaking. The next day, the cell lysates were incubated with protein A/G magnetic beads at 4 °C for 2 h. Thereafter, the immunoprecipitated complexes were washed twice with IP lysis buffer. After being boiled in 5×SDS loading buffer for 10 min, the samples were subjected to western blot analysis.

### Immunofluorescence (IF)

Briefly, cells were seeded on the Lab-Tek chamber slides at a density of 5 × 10^3^ and cultured for 24 h. Subsequently, cells were fixed with 4% paraformaldehyde and permeabilized with 0.5% TritonX-100. After being washed with PBS, the slides were incubated with primary antibodies targeting E-cadherin, N-cadherin, and Vimentin overnight at 4 °C in a humidified chamber. The next day, the glass slides were washed twice with PBS, and were then incubated with corresponding second antibodies for 1 h at room temperature in dark. The nuclei were stained with DAPI solution (1 µg/ml) before being observed and photographed using a fluorescence microscopy.

### Cycloheximide (CHX) chase

786-O cells stably knocking down or overexpressing TRIM26 and their corresponding control cells were treated with CHX (25 µg/mL), and cells were then harvested for indicated time point (0, 2, 4, 8 h). Cell lysates were obtained using RIPA lysis buffer and were subsequently subjected to western blot assay to observe the half-life of ETK.

### In vivo ubiquitination assay

786-O cells stably knocking down or overexpressing TRIM26 and their corresponding control cells were exposed to MG132 (20 µM) for 6 h before being harvested. Total proteins were extracted using RIPA lysis buffer and then incubated with antibody targeting ETK overnight at 4 °C with rotation. The next day, the immune-precipitates were used for western blot analysis to detect ubiquitination of endogenous ETK.

### Animal studies

The four-weeks old male BALB/c-nu mice, obtained from HFK Bioscience (Beijing, China), were raised in the specific pathogen-free (SPF) animal laboratory of the Animal Center of Wuhan University Renmin Hospital. Mice were randomly divided into two groups, with 6 mice per group. 786-O cells stably overexpression TRIM26 (5 × 10^6^ cells suspended in 100 µl PBS) or control cells were injected into nude mice to generate subcutaneous tumors. Tumor volumes were monitored every week by measuring the length (a) and width (b) of tumors, and were calculated using the following formula: *V*(mm^3^) = 1/2*a*b^2. Thirty-five days post-injection, the mice were sacrificed by the cervical dislocation method. The tumor tissues were isolated and photographed, tumor weights were also measured. Subsequently, tumors were fixed with 4% paraformaldehyde or stored in -80 °C for the purpose of further experiments.

### Immunohistochemistry (IHC)

Briefly, fresh tissues were fixed in 4% paraformaldehyde for 24 h and then embedded in paraffin, followed by being cut into 5 μm sections. Then, the slides were deparaffinized in a solution of xylene and rehydrated with graded ethanol solutions. Next, antigens were retrieved by boiling the slides in a 0.01 M sodium citrate (pH 6.0) buffer for 30 min. Then, sections were then sealed with 3% hydrogen peroxide for 10 min, followed by being blocked with 5% BSA for 1 h at room temperature. Subsequently, immunohistochemistry staining of specimens were performed by incubating sections with specific antibodies including anti-TRIM26 (Cat: sc-393,832, Santa Cruz Biotechnology), anti-p-AKT (Cat: #4060, Cell Signaling Technology), and anti-p-mTOR (Cat: #5536, Cell Signaling Technology), in humidified chamber at 4 °C overnight. The next day, the sections were incubated with streptavidin-perosidase-conjugated second antibody at appropriate concentrations for 1 h. The immune complexes were visualized using a DBA kit (Servicebio Technology, Wuhan, China). Finally, the slides were counterstained with haematoxylin before being observed and photographed under a light microscope. The staining intensity and the proportion of positive tumor cells were evaluated by two independent experienced examiners to compare relative protein expression levels in different groups.

### Statistically analysis

All the experiments were conducted in at least three biological repeats and the data were showed as Mean ± Sem. All the statistically analysis was performed using R 4.1.0 and GraphPad Prism 8.0. Statistical significance was calculated using student’s t-test or one-way ANOVA to compare differences between different groups. *P*. value of less than 0.05 was considered as significant difference.

## Results

### TRIM26 was down-regulated in ccRCC and associated with patient survival

**Fig. 1:**
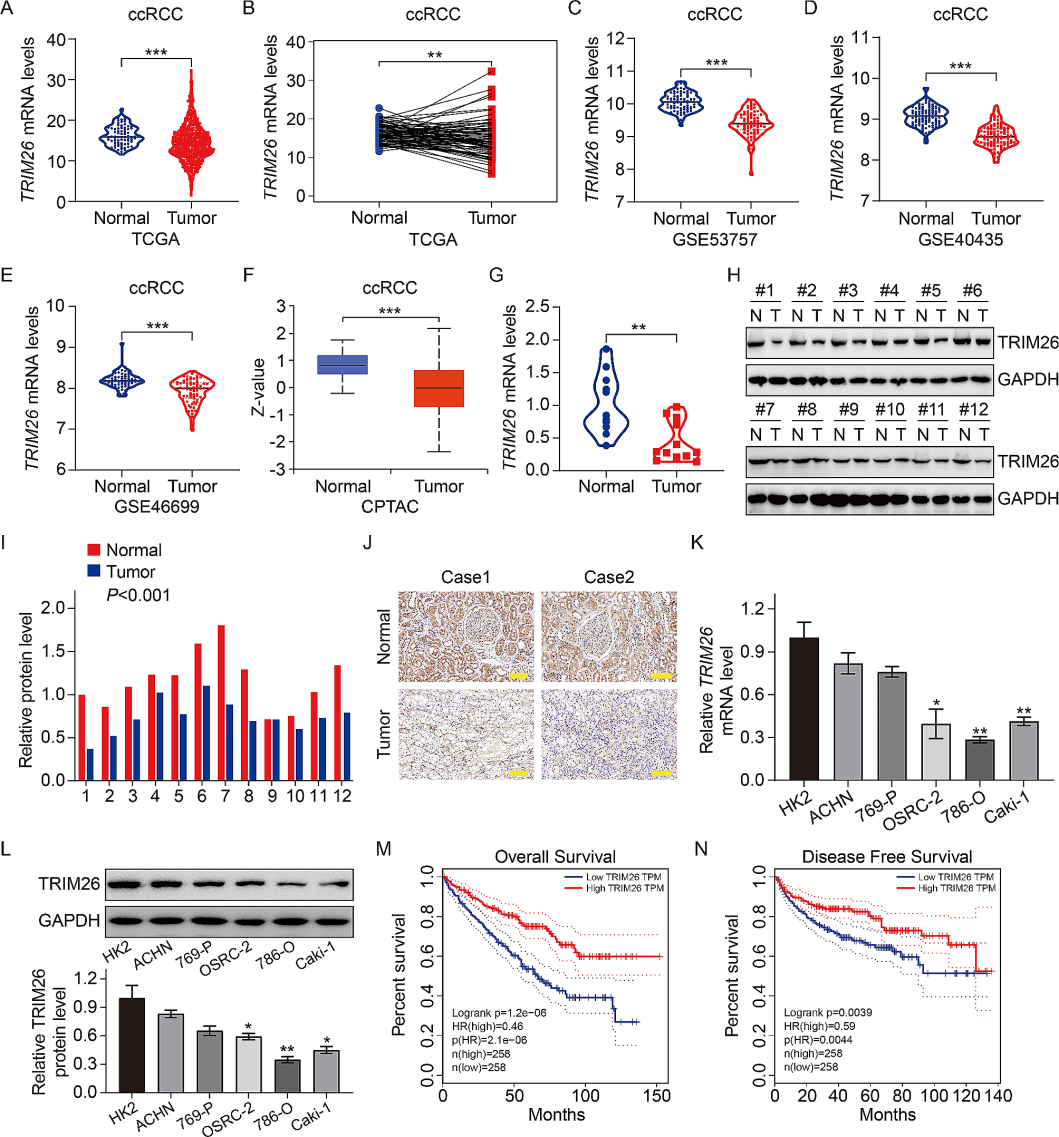
TRIM26 was downregulated in ccRCC and associated with patient’s prognosis. (**A**) Comparison of TRIM26 mRNA expression between normal and tumor samples. (**B**) TRIM26 expression in ccRCC tissues and normal renal tissues. (**C**) Comparison of TRIM26 expression in ccRCC tissues and matched normal tissues. (**D-F**) TRIM26 expression in ccRCC tissues and normal tissues in the three independent GEO datasets (GSE53757, GSE40435, and GSE46699). (**G**) Comparison of TRIM26 protein level in ccRCC tissues and normal tissues. (**H**) qRT-PCR analysis of TRIM26 expression in ccRCC tissues and adjacent normal tissues. (**I-J**) Western blot analysis of TRIM26 level in ccRCC tissues and adjacent normal tissues, and quantitative analysis. (**K**) IHC analysis of TRIM26 expression in ccRCC tissues and normal tissues. Scale bar, 100 μm. (**L-M**) qRT-PCR and western blot analyses of TRIM26 levels in ccRCC cells (ACHN, 769-P, OSRC-2, 786-O, Caki-1) and normal renal cell line (HK-2). (**N-O**) Analyzing the association of TRIM26 expression with patients’ overall survival and diseases free survival. *P < 0.05, **P < 0.01, ***P < 0.001

In order to determine the significance of TRIM26 in the development of ccRCC, we first evaluated the expression of TRIM26 in ccRCC tissues and normal renal tissues using data obtained from public resources. The findings indicated that the mRNA expression of TRIM26 was markedly reduced in ccRCC tissues compared to normal tissues (Fig. 1A). Furthermore, a comparable outcome was achieved when comparing the expression of TRIM26 in ccRCC tissues and matched adjacent non-cancerous renal tissues (Fig. 1B). The decreased expression of TRIM26 in ccRCC was also validated by three separate GEO datasets (GSE53757, GSE40435, and GSE46699) (Fig. 1C-E). Consistent with mRNA expression, significantly diminished TRIM26 protein expression was observed in ccRCC tissues according to the UALCAN database (Fig. 1F). In addition, we conducted qRT-PCR, western blot and IHC staining assays to examine TRIM26 expression in 12 paired fresh ccRCC and neighboring normal renal tissues. The findings indicated a significant decrease in both the mRNA and protein levels of TRIM26 in ccRCC (Fig. 1G-J). We also examined endogenous TRIM26 expression levels in several ccRCC cell lines (ACHN, 769-P, OSRC-2, 786-O, Caki-1) as well as in normal renal cell line (HK-2). The findings indicated a considerable reduction in the mRNA and protein levels of TRIM26 in ccRCC cells (OSRC-2, 786-O, Caki-1) compared to HK-2 cells, and slight reduction of TRIM26 in ACHN and 769-P cells (Fig. 1K-L). The Kaplan-Meier analysis demonstrated that those with high expression of TRIM26 had a notably extended overall survival and disease-free survival in comparison to those with low expression of TRIM26 (Fig. 1M-N). In addition, the findings of both univariate and multivariate Cox regression analysis indicated that the expression of TRIM26 was a protective factor and an independent prognostic predictor in patients with ccRCC (Table 2).

**Table 2:** Univariable and multivariable analysis of the TRIM26 expression and clinical factors in the TCGA cohort

Variables	Univariable analysis				Multivariable analysis			
	HR	95% CI of HR		*P*	HR	95% CI of HR		*P*
		Lower	Upper			Lower	Upper	
Age (≤ 60 vs. > 60)	1.79	1.31	2.44	0.00	1.61	1.17	2.21	0.00
Gender (Female vs. Male)	0.93	0.68	1.27	0.65	0.80	0.57	1.12	0.19
Grade (I/II vs. III/IV)	2.59	1.84	3.66	0.00	1.73	1.19	2.50	0.00
Stage (I/II vs. III/IV)	3.61	2.62	4.98	0.00	2.13	1.03	4.42	0.04
T (T 1/2 vs. T 3/4)	3.00	2.21	4.09	0.00	0.91	0.49	1.72	0.78
M (M0 vs. M1)	4.20	3.07	5.76	0.00	2.41	1.63	3.56	0.00
TRIM26 (High vs. Low)	0.96	0.92	0.99	0.02	0.95	0.91	0.99	0.01

### TRIM26 overexpression inhibited malignant behaviors in ccRCC cells

**Fig. 2:**
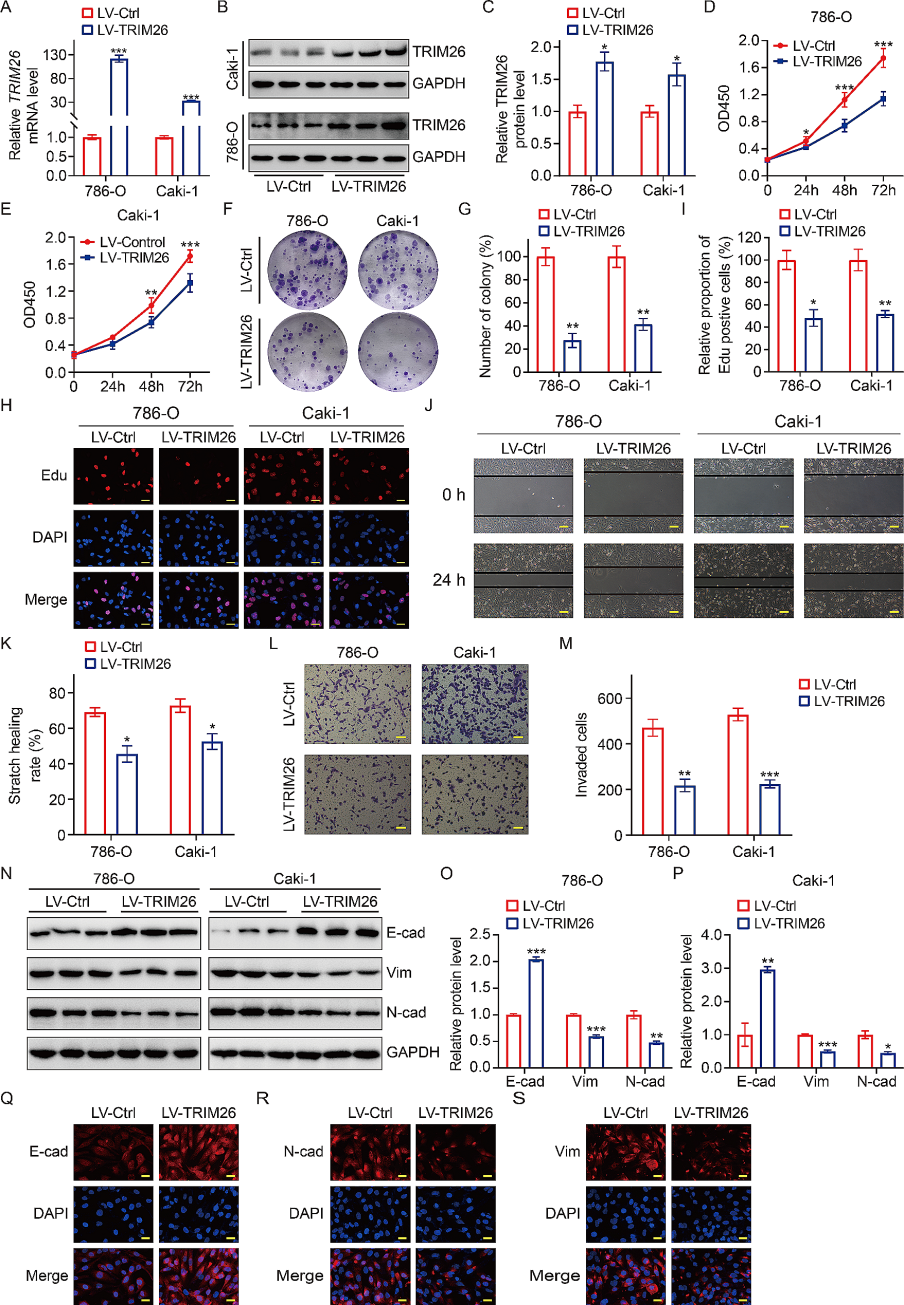
Overexpression of TRIM26 inhibited ccRCC cell proliferation, migration, invasion, and EMT. (**A-C**) TRIM26 expression levels in the indicated ccRCC cells were determined by qRT-PCR and western blot assays. (**D-E**) CCK-8 assay was performed in 786-O and Caki-1 cells to evaluate cell proliferation ability. (**F-G**) Colony formation assay showed the effect of TRIM26 overexpression on long time cell proliferation of 786-O and Caki-1 cells. (**H-I**) Edu incorporation assay was utilized to assess cell proliferation in 786-O and Caki-1 cells stably overexpressing TRIM26. Scale bar, 200 μm. (**J-K**) The wound healing assay evaluated cell migration ability after overexpressing TRIM26, and quantitative analysis. Scale bar, 200 μm. (**L-M**) The transwell invasion assay evaluated cell invasion ability after overexpressing TRIM26, and quantitative analysis. Scale bar, 200 μm. (**N-P**) Western blot assay was conducted to detect expression levels of EMT-related proteins including E-cad, N-cad, and Vim, and quantitative analysis. (**Q-S**) Immunofluorescence staining to evaluate the expression levels of E-cad, N-cad, and Vim in 786-O cells stably overexpressing TRIM26. Scale bar, 200 μm. *P < 0.05, **P < 0.01, ***P < 0.001

Having established the low expression status of TRIM26 in ccRCC, we will now investigate the impact of TRIM26 overexpression on the phenotypes of ccRCC cells. We constructed ccRCC cell lines (786-O, Caki-1) with stable TRIM26 overexpression through a lentivirus infection approach. Consistent with expectations, the mRNA and protein levels of TRIM26 were significantly elevated in clear cell renal cell carcinoma (ccRCC) cells that were infected with LV-TRIM26, in comparison to the control cells (Fig. 2A-C). The impact of alterations in TRIM26 expression on the proliferation of ccRCC cells was assessed by the implementation of CCK-8, colony formation, and EdU incorporation studies. The findings demonstrated a significant decrease in the optical density value (Fig. 2D-E), the number of colonies (Fig. 2F-G), and the presence of EdU positive cells (Fig. 2H-I) after the overexpression of TRIM26. These results strongly show that TRIM26 overexpression negatively affected the growth capacity of ccRCC cells. Furthermore, wound healing and transwell assays were conducted to assess the impact of TRIM26 on cell migration and invasion. The findings demonstrated that overexpression of TRIM26 significantly reduced the migratory (Fig. 2J-K) and invasive (Fig. 2L-M) abilities of 786-O and Caki-1 cells. Epithelial-mesenchymal transition (EMT) is a significant process that involves changes in cell phenotype and contributes to the ability of cancer cells to migrate and invade surrounding tissues [26]. In this study, we investigated the role of TRIM26 in the EMT process of ccRCC cells by examining the expression of classic markers such as E-cadherin, N-cadherin, and Vimentin. Consistent with phenotypic changes caused by modified TRIM26 expression, western blot experiments demonstrated that TRIM26 overexpression led to an increase in the expression of the epithelial marker E-cadherin, while simultaneously decreasing the expression of the mesenchymal cell markers N-cadherin and Vimentin (Fig. 2N-P). Immunostaining analysis conducted on 786-O cells indicated a rise in the levels of E-cadherin, as well as a decrease in the staining intensity of N-cadherin and Vimentin, after the overexpression of TRIM26 (Fig. 2Q-S). The studies suggest that TRIM26 functions as a tumor suppressor gene, inhibiting cell proliferation, migration, invasion, and epithelial-mesenchymal transition.

### Silencing of TRIM26 accelerated cell proliferation, migration, and invasion and promoted EMT in ccRCC

**Fig. 3:**
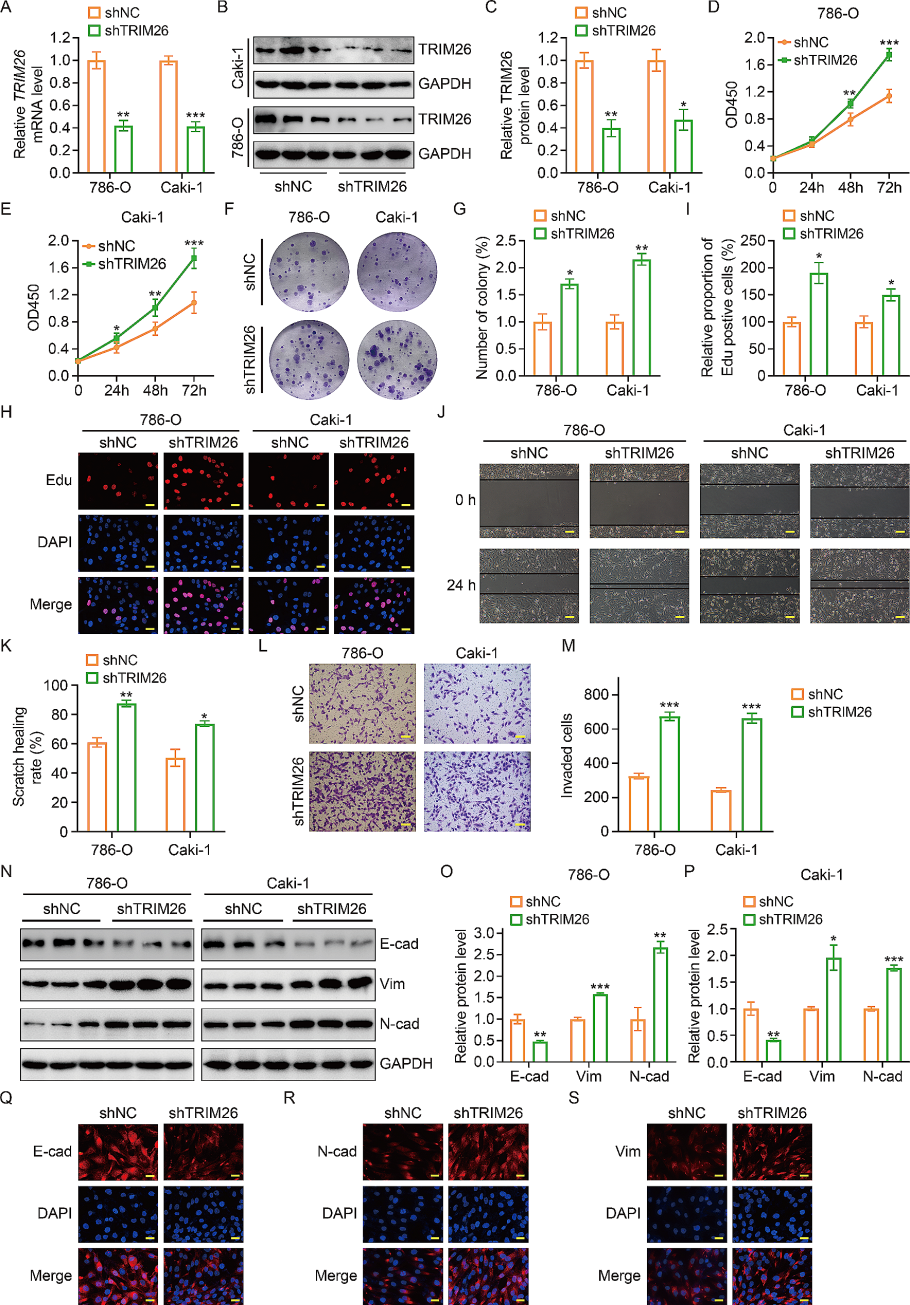
Silence of TRIM26 promoted ccRCC cell proliferation, migration, invasion, and EMT. (**A-C**) qRT-PCR and western blot assays confirmed the silence of TRIM26 in the indicated ccRCC cells. (**D-E**) CCK-8 assay was performed in 786-O and Caki-1 cells stably knocking down of TRIM26 to evaluate cell proliferation ability. (**F-G**) Colony formation assay showed the effect of TRIM26 knockdown on long time cell proliferation of ccRCC cells. (**H-I**) Edu incorporation assay was conducted to evaluate cell proliferation ability in ccRCC cells stably knocking down of TRIM26. Scale bar, 200 μm. (**J-K**) The wound healing assay evaluated cell migration ability after silencing TRIM26, and quantitative analysis. Scale bar, 200 μm. (**L-M**) The transwell invasion assay evaluated cell invasion ability after silencing TRIM26, and quantitative analysis. (**N-P**) Western blot assay was performed to investigate protein levels of E-cad, N-cad, and Vim, and quantitative analysis. (**Q-S**) Immunofluorescence staining to evaluate the expression levels of E-cad, N-cad, and Vim in 786-O cells stably knocking down of TRIM26. Scale bar, 200 μm. *P < 0.05, **P < 0.01, ***P < 0.001

In order to assess the specific functions of TRIM26 in the proliferation, migration, and invasion of ccRCC cells, we conducted a series of functional tests using ccRCC cell lines that had been modified to have reduced levels of TRIM26. The lentivirus containing shRNA that selectively targets TRIM26 was assessed for its knockdown effectiveness using qRT-PCR and western blot tests (Fig. 3A-C). The CCK-8 test results demonstrated enhanced cell proliferation rates in 786-O and Caki-1 cells after the suppression of TRIM26 (Fig. 3D-E). The results from colony formation and EdU incorporation experiments demonstrated that the suppression of TRIM26 led to a higher number of colonies and an increased presence of EdU positive cells (Fig. 3F-I). This provides additional evidence of enhanced cell proliferation in ccRCC cells after the silencing of TRIM26. During the wound healing experiment, it was shown that cells with reduced levels of TRIM26 healed their wounds nearly entirely after 24 h. In contrast, control cells only closed roughly 40–50% of the wound width (Fig. 3J-K). This indicates that silencing TRIM26 leads to enhanced closure of wound regions. The transwell invasion experiment demonstrated that the depletion of TRIM26 resulted in a substantial increase in the quantity of cells that invaded through the matrigel-coated membrane (Fig. 3L-M). Furthermore, the suppression of TRIM26 in ccRCC cells led to a considerable increase in the expression of N-cadherin and Vimentin, while inhibiting the expression of E-cadherin, as confirmed by western blot assay (Fig. 3N-P) and immunostaining assay (Fig. 3Q-S). Overall, the findings clearly demonstrated that suppressing TRIM26 significantly increased the cell’s ability to proliferation, migration and invasion. Additionally, TRIM26 was shown to play a crucial role in inhibiting the process of epithelial-mesenchymal transition in ccRCC.

#### Transcriptome analysis identified mTOR signaling as the downstream pathway of TRIM26

**Fig. 4:**
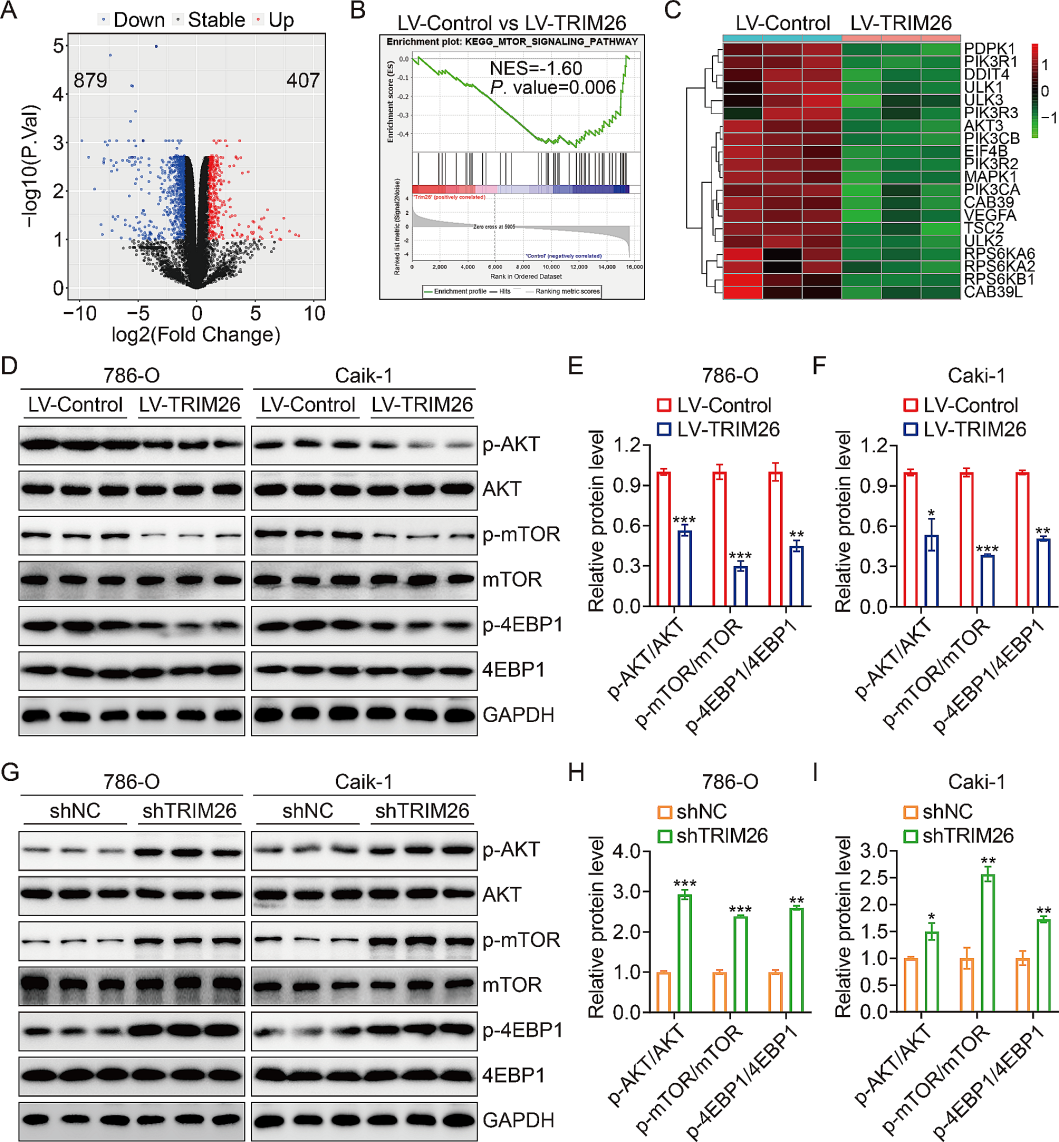
Transcriptome analysis identified mTOR signaling as the downstream pathway of TRIM26. (**A**) Comparison of the differentially expressed genes between LV-Control and LV-TRIM26 groups. (**B**) GSEA exhibiting the enrichment of mTOR-related gene signatures. (**C**) Heatmap showing the expression levels of genes in mTOR signaling pathway in LV-Control and LV-TRIM26 groups. (**D-F**) Protein expression levels of p-AKT, AKT, p-mTOR, mTOR, p-4EBP1, and 4EBP1 in 786-O and Caki-1 cells infected with lentivirus overexpressing TRIM26 or empty control vector, and quantitative analysis. (**G-I**) Protein expression levels of p-AKT, AKT, p-mTOR, mTOR, p-4EBP1, and 4EBP1 in 786-O and Caki-1 cells infected with lentivirus containing shTRIM26 or shNC, and quantitative analysis. *P < 0.05, **P < 0.01, ***P < 0.001

To delineate the functional implication of TRIM26 in ccRCC, we conducted transcriptome analysis using high-throughput RNA sequencing (RNA-Seq) on 786-O cells with TRIM26 overexpression or control. Figure 4A demonstrates that 407 differentially expressed genes (DEGs) were increased in expression, whereas 879 DEGs were decreased in expression in TRIM26-upregulated 786-O cells. Following that, a gene set enrichment analysis was performed, revealing a strong correlation between the expression of TRIM26 and the suppression of mTOR signaling (Fig. 4B-C). This suggests that TRIM26 may play a regulatory function in the transduction cascade of mTOR signaling. Hence, we conducted a more detailed analysis to determine the impact of TRIM26 overexpression or knockdown on the mTOR signaling pathway in ccRCC by examining the phosphorylation levels of upstream kinase AKT and key mTOR pathway components such as p-mTOR and p-4EBP1. The Western blotting experiment findings showed a substantial drop in the expression levels of p-AKT, p-mTOR, and p-4EBP1 in 786-O and Caki-1 cells with TRIM26 overexpression. However, the total protein levels of AKT and mTOR remained unaltered (Fig. 4D-F). In contrast, the absence of TRIM26 led to elevated levels of p-AKT, p-mTOR, and p-4EBP1 expression in ccRCC cells (Fig. 4G-I). Furthermore, the compound LY294002, which inhibits the AKT/mTOR signaling pathway, was able to reverse the enhanced proliferation, migration, and invasion capabilities produced by the suppression of TRIM26 in ccRCC cells. Collectively, our findings so far demonstrate that TRIM26 hinders the advancement of ccRCC by deactivating the AKT/mTOR signaling pathway (Supplementary Fig. 1A-F).

### TRIM26 binds to and degrades ETK

**Fig. 5:**
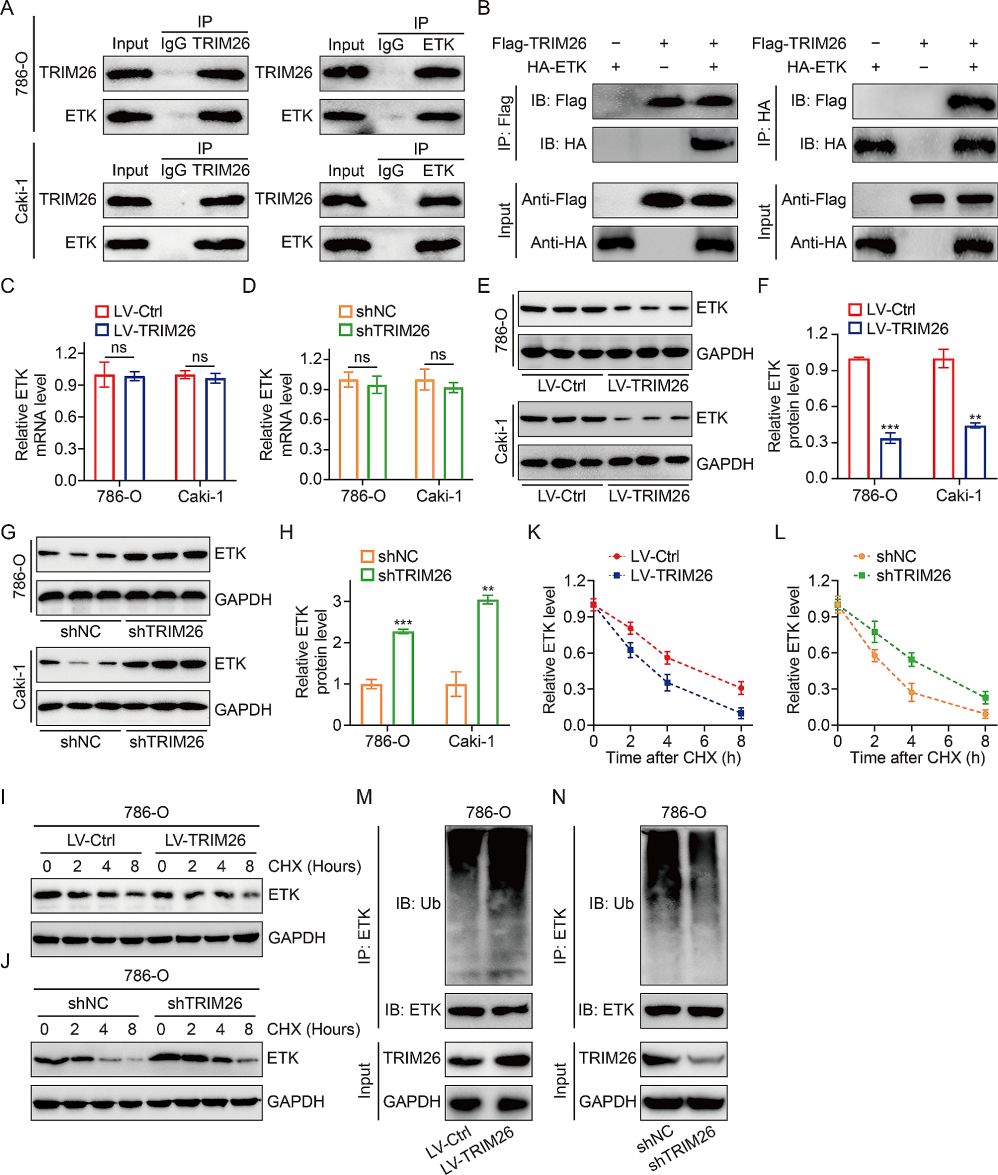
TRIM26 binds to and degrades ETK. (**A**) The endogenous co-immunoprecipitation of TRIM26 and ETK in 786-O and Caki-1 cells. (**B**) The co-immunoprecipitation of FLAG-TRIM26 and HA-ETK in 293T cells. (**C-D**) The effect of TRIM26 overexpression or knockdown on mRNA levels of ETK. (**E-F**) ETK protein levels in cells stably overexpressing TRIM26 and control cells, and quantitative analysis. (**G-H**) ETK protein levels in cells stably knocking down of TRIM26 and control cells, and quantitative analysis. (**I-L**) 786-O cells stably overexpressing or knocking down of TRIM26, and their corresponding control cells were treated with CHX (50 µg/ml) for indicated time, and ETK protein levels were detected by western blot assay. (**M-N**) In vitro ubiquitination assay was conducted to detect the ubiquitination of ETK after overexpressing or knocking down of TRIM26 in 786-O cells. *P < 0.05, **P < 0.01, ***P < 0.001

In order to elucidate the fundamental mechanism by which TRIM26 interacts with mTOR signaling, we searched for TRIM26-binding proteins based on prior studies. ETK, a member of the TEC family of non-receptor tyrosine kinases, has caught our interest due to its reported interaction with TRIM26 and its role as an upstream kinase of AKT. Thus, we conducted immunoprecipitation tests to validate the interaction between TRIM26 and ETK. The inherent physical interaction between the two proteins was verified in the lysates of 786-O and Caki-1 cells (Fig. 5A). Subsequently, in HEK 293T cells, immunoprecipitation with anti-FLAG or anti-HA antibodies brought down HA-tagged ETK or Flag-tagged TRIM26, demonstrating an interaction between the two tagged proteins (Fig. 5B). However, the impact of this interaction on the physiological function of ETK remains uncertain. In order to investigate this inquiry, we first identified the protein and mRNA concentrations of ETK in ccRCC cells, since TRIM26 served as an E3 Ub ligase responsible for the degradation of downstream targets. The qRT-PCR and western blot assays demonstrated that altering the expression of TRIM26 did not affect the mRNA levels of ETK (Fig. 5C-D). However, it did lead to a decrease or increase in the protein levels of ETK (Fig. 5E-H). This suggests that TRIM26 exerts a negative regulatory effect on the protein levels of ETK at the post-transcriptional level. We then examined the influence of TRIM26 on the stability of ETK by the implementation of a cycloheximide (CHX) test. The findings demonstrated that overexpression of TRIM26 expedited the degradation of ETK, whereas the suppression of TRIM26 extended the duration of ETK in 786-O cells (Fig. 5I-L). This suggests that TRIM26 likely enhances the degradation of ETK via the ubiquitin-proteasome pathway. Hence, we conducted an in vivo ubiquitination experiment and observed that the levels of ubiquitination of ETK were elevated in 786-O cells that were overexpressing TRIM26, as shown in Fig. 5M. Consistent with this discovery, the absence of endogenous TRIM26 resulted in the elimination of ETK ubiquitination (Fig. 5N). Our findings indicate that the E3 ubiquitin ligase TRIM26 reduces the levels of ETK by promoting its degradation via ubiquitination and proteasomal pathways.

### TRIM26 inhibited cell malignancy via ETK-mediated inactivation of mTOR signaling pathway

**Fig. 6:**
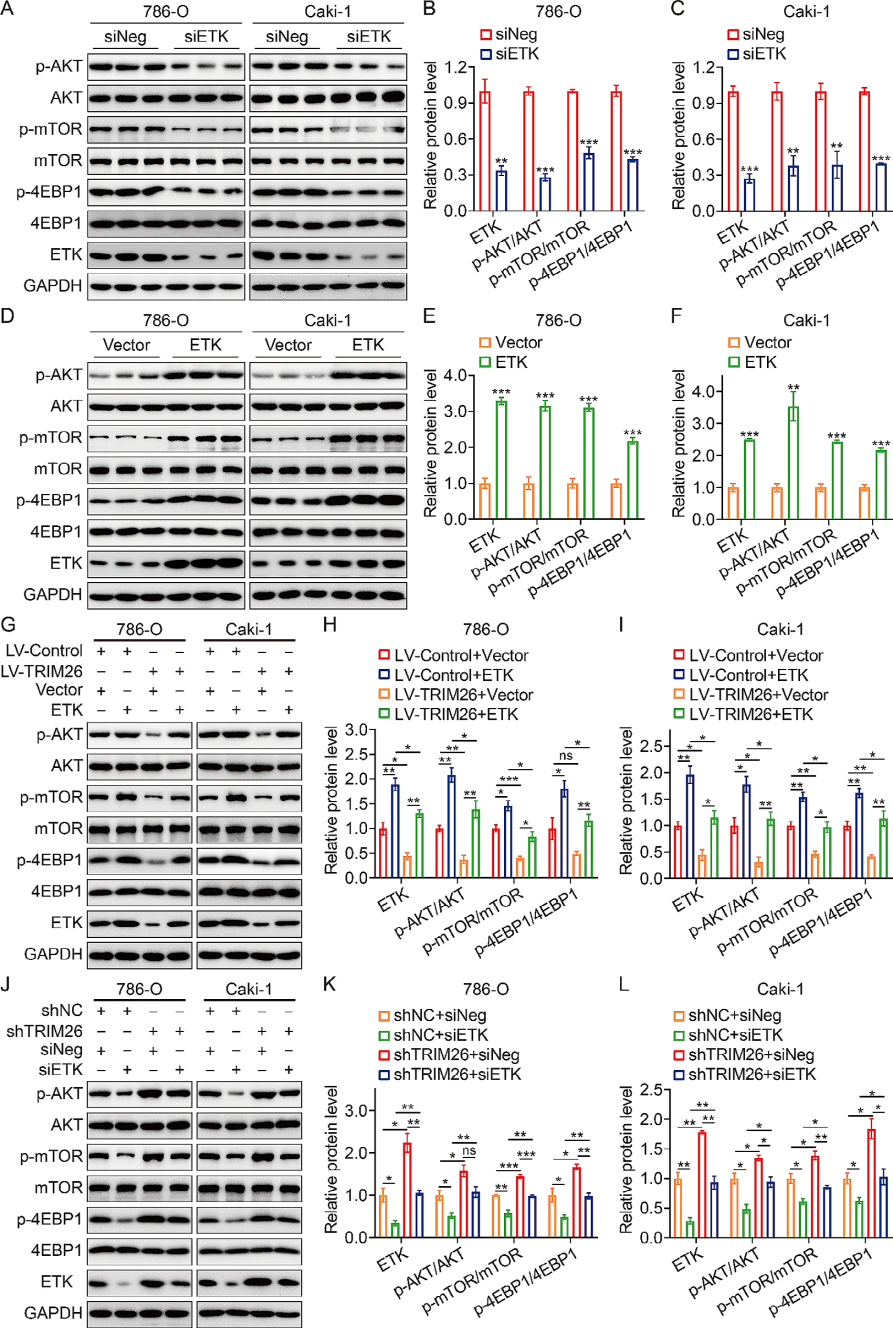
ETK mediated the effect of TRIM26 on AKT/mTOR signaling pathway. (**A-C**) Western blot showed the phosphorylation level of AKT, mTOR, and 4EBP1 after silencing ETK, and quantitative analysis. (**D-F**) Western blot showed the protein levels of AKT, p-AKT, mTOR, p- mTOR, 4EBP1, and p-4EBP1 when the ETK was overexpressed, and quantitative analysis. (**G-I**) Western blot was performed to evaluate the phosphorylation levels of AKT, mTOR, and 4EBP1 in TRIM26-upregulated cells after transfection of ETK overexpression plasmid and empty vector, and quantitative analysis. (**J-L**) Evaluation of the protein levels of AKT, p-AKT, mTOR, p- mTOR, 4EBP1, and p-4EBP1 in TRIM26-silenced cells transfected with siETK or siNeg. *P < 0.05, **P < 0.01, ***P < 0.001

In order to ascertain the inhibitory effect of TRIM26 on AKT/mTOR signaling via ETK, we first examined the impact of ETK on the activation of AKT/mTOR signaling by quantifying the amounts of p-AKT, p-mTOR, and p-4EBP1 proteins. The Western blot experiment demonstrated that the inhibition of ETK resulted in a significant reduction in the expression of ETK, p-AKT, p-mTOR, and p-4EBP1 in 786-O and Caki-1 cells (Fig. 6A-C). In contrast, the overexpression of ETK in ccRCC cells resulted in the observed up-regulation of these proteins (p-AKT, p-mTOR, and p-4EBP1) (Fig. 6D-F). Subsequently, we altered the expression of ETK in 786-O and Caki-1 cells stably overexpressing or knocking down of TRIM26, and their corresponding control cells. Immunoblotting analysis revealed that the increased expression of ETK reinstated the levels of phosphorylation in AKT, mTOR, and 4EBP1 in ccRCC cells that were stably overexpressing TRIM26 (Fig. 6G-I). Furthermore, the intervention of ETK reduced the increase in p-AKT, p-mTOR, and p-4EBP1 caused by TRIM26 knockdown (Fig. 6J-L). Collectively, our findings clearly indicate that TRIM26 modulates mTOR signaling via ETK. To further investigate whether TRIM26 inhibited cell malignant behaviors depending on ETK-mediated inactivation of mTOR signaling pathway, we altered the expression levels of ETK and TRIM26 in ccRCC cells at the same time and examined their impact on malignant behavior. The CCK-8 experiment demonstrated that TRIM26 inhibited cell growth in 786-O and Caki-1 cells, and the overexpression of ETK may partly counteract this effect (Supplementary Fig. 2A-B). In addition, the suppression of ETK hindered the proliferation of ccRCC cells induced by the inhibition of TRIM26 (Supplementary Fig. 2C-D). Furthermore, wound healing and transwell invasion assays revealed that the overexpression of ETK reversed the inhibitory effects of TRIM26 overexpression on cell migration and invasion abilities (Supplementary Fig. 2E, 2G). Conversely, the knockdown of ETK reversed the enhanced cell migration and invasion induced by TRIM26 silence (Supplementary Fig. 2F, 2 H).

### Effect of TRIM26 overexpression on ccRCC cell growth in vivo

**Fig. 7:**
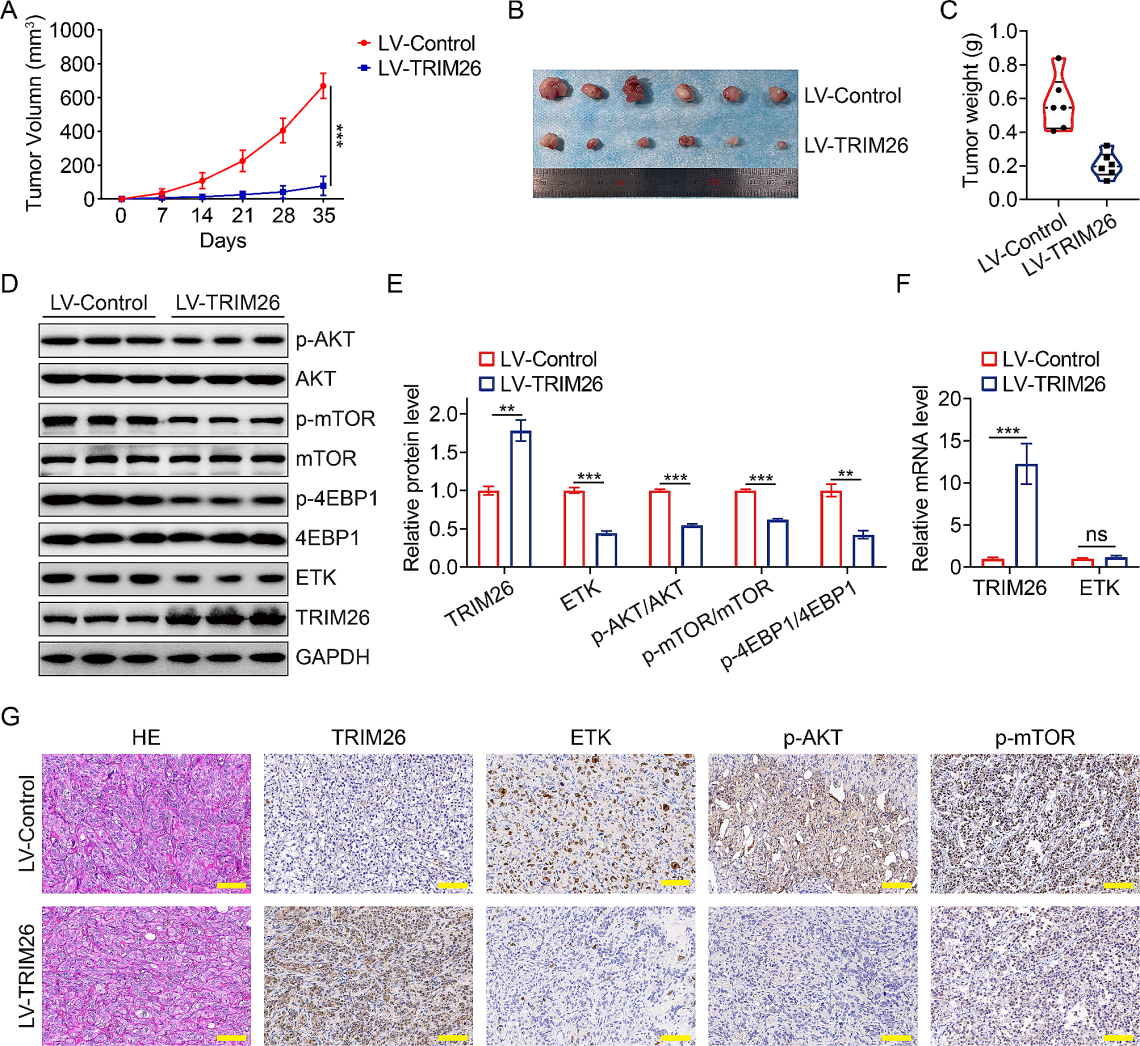
Overexpression of TRIM26 inhibited ccRCC growth in vivo. (**A**) Tumor volumes were monitored every week until 35 days post-injection. (**B-C**) The tumors of the two groups were isolated and photographed after the mice were sacrificed, and the tumors’ weight were measured. (**D-E**) The protein levels of TRIM26, ETK, p-AKT, p-mTOR, and p-4EBP1 in tumor tissues were detected by western blot assay. (**F**) qRT-PCR experiment was performed to evaluate the effect of TRIM26 overexpression on ETK mRNA level. (**G**) IHC demonstrated the expression levels of TRIM26, ETK, p-AKT, p-mTOR. Scale bar, 200 μm. *P < 0.05, **P < 0.01, ***P < 0.001

To identify the exact role of TRIM26 on ccRCC cell growth in *vivo*, we created xenograft models by subcutaneously inoculating 786-O cells stably overexpressing TRIM26 and control cells. We then observed and measured the growth of the tumors on a weekly basis. The tumor growth curve demonstrated a significantly reduced rate of growth in the LV-TRIM26 groups compared to the LV-Ctrl group (Fig. 7A). In addition, the LV-TRIM26 group exhibited significantly less tumor size and weight compared to the LV-Ctrl group, as seen in Fig. 7B-C. The western blot experiment verified an elevation in the TRIM26 protein level in the tumor tissues of the LV-TRIM26 groups. Additionally, there was a reduction in the protein levels of ETK, p-AKT, p-mTOR, and p-4EBP1 (Fig. 7D-E). According to the qRT-PCR experiment, the LV-TRIM26 group showed a rise in the TRIM26 mRNA level compared to the LV-Ctrl group. However, there was no change in the ETK mRNA level (Fig. 7F). Consistent with the aforementioned results, immunohistochemistry staining showed an increase in TRIM26 expression, and a decrease in ETK, p-AKT, and p-mTOR expression in the LV-TRIM26 group compared to the LV-Ctrl group (Fig. 7G). Collectively, our findings suggest that the increased expression of TRIM26 hinders the development of cells by disrupting ETK, leading to the deactivation of AKT/mTOR signaling in ccRCC.

## Discussion

There was an increasing number of studies suggested that TRIM family proteins are dysregulated in several types of cancer and may have either a protective or promotive impact on the development and progression of cancer [27–29]. Overall, alterations in the manifestation of TRIM proteins have a significant impact on controlling malignant activities in tumors, such as cell growth, metastasis, stem cell characteristics, and resistance to chemotherapy. These effects may occur either via the involvement of E3 ligases activity or independently of them [30–33]. In renal cell carcinoma, multiple TRIM proteins had been reported to be associated with cancer development and prognosis. For instance, TRIM7 was identified as a tumor suppressor in ccRCC by decreasing the levels of Src protein via the ubiquitin-proteasome pathway, hence exerting a negative regulatory effect on HIF-1 signaling [34]. The expression of TRIM21 was significantly reduced in renal cell carcinoma, and this decreased expression was linked to unfavorable clinicopathological features and decreased overall survival in RCC patients. Additional investigation uncovered that TRIM21 hindered cell proliferation and metastasis by facilitating the breakdown of HIF-1α, hence suppressing cell glycolysis in renal cancer cells [35]. We recently developed a prognostic signature for clear cell renal cell carcinoma (ccRCC) using genes from the TRIM family. Additionally, we found that some TRIM proteins, such as TRIM26, had predictive value in ccRCC [36]. Nevertheless, the specific function of TRIM26 in ccRCC has not been previously documented.

In this work, we have shown that the expression of TRIM26 was reduced in ccRCC tissues compared to nearby normal renal tissues through several independent public resources and further experimental validation. Meanwhile, we discovered that decreased TRIM26 expression was associated with worse overall survival and diseases-free survival in ccRCC patients, suggesting that TRIM26 may serve as a predictive biomarker. It is necessary to gather a substantial real-world group of individuals in order to conduct a more in-depth investigation into the ability of TRIM26 and other TRIM family proteins to predict the performance of ccRCC. Furthermore, we altered the expression of TRIM26 using lentiviral infection and examined its impact on the malignant behaviors of ccRCC cells. Our empirical investigations shown that enforced expression of TRIM26 impeded the proliferation, migration, invasion, and epithelial-mesenchymal transition process in ccRCC cells. Conversely, the suppression of TRIM26 had the contrary impact. Furthermore, we have verified the suppressive impact of TRIM26 on ccRCC in an in vivo setting. The data together revealed that TRIM26 plays a function as a tumor suppressor in the carcinogenesis and development of ccRCC.

In order to better understand how TRIM26 suppresses malignant behaviors in ccRCC, we conducted RNA-seq followed by bioinformatic analysis. Our findings revealed a strong correlation between the upregulation of TRIM26 expression and the inhibition of mTOR signaling. This suggests that TRIM26 may play a regulatory role in the transduction cascade of mTOR signaling, which is commonly disrupted in human cancer [37]. The mammalian target of rapamycin (mTOR) is a well-preserved protein kinase that specifically acts on serine and threonine amino acid residue. It is classified as a member of the phosphoinositide 3-kinase-related kinase (PIKK) family. The mTOR forms two structurally and functionally distinct multiprotein complexes, naming mTORC1 and mTORC2 [38], and they plays a crucial role in regulating cell proliferation and growth rate by reacting to various stimuli, such as growth hormones, nutrition, and energy [39, 40]. The upstream components of the mTOR pathway include a variety of signaling molecules, making it involved in several signaling pathways in mammals. One of the most significant routes is the Akt-mTOR signaling system [41, 42]. Multiple studies have shown that cancers often exhibit excessive activation of the AKT/mTOR signaling pathway. This heightened activation further stimulates the growth of tumor cells, as well as their local invasion and spread to healthy tissues in distant locations [43, 44]. Consequently, it is now a prominent focus in the field of anti-tumor therapeutic investigation [45]. Through using the high-throughput sequencing method, we have discovered a potential correlation between the overexpression of TRIM26 and the inhibition of mTOR. Following this, we verified that TRIM26 exerted a negative regulatory role on the AKT/mTOR signaling pathway in ccRCC. This was shown by a reduction in the expression of p-AKT and p-mTOR with TRIM26 overexpression, and an elevation in p-AKT and p-mTOR levels when TRIM26 was suppressed. Collectively, we may infer that TRIM26 suppressed cell growth and invasion in ccRCC via deactivating the AKT/mTOR pathway.

Ubiquitination is an essential cellular process that dynamically regulates certain proteins involved in cell growth, survival, and other functions, hence maintaining cell homeostasis. The traditional ubiquitination cascade consists of three enzymes: the E1 Ub-activating enzyme, the E2 Ub-conjugating enzyme, and the E3 Ub ligase. The E3 Ub ligase is responsible for identifying the particular substrate protein and facilitating the transfer of Ub from E2 to the substrate [46]. Hence, the E3 Ub ligase is the crucial element of the ubiquitination cascade. TRIM26 has been identified as a prototypical E3 Ub ligase and functions in a ubiquitination-dependent way in several types of cancer [21, 47]. Thus, we tried to identify the specific downstream substrate of TRIM26 in ccRCC. ETK, a non-receptor tyrosine kinase, caught our interest due to its identification as an interacting protein of TRIM26 in prior research [48] and our immunoprecipitation findings. Additionally, it has the potential to operate as an upstream kinase of AKT.

ETK, often referred to as bone marrow tyrosine kinase (BMX), has been implicated in playing a crucial regulatory function in the development of tumors [49–51]. An abnormal expression of ETK has been detected in several cancer types, and it typically plays a more prominent role in promoting cancer growth [52, 53]. In RCC, the expression of ETK was significantly higher in tumor tissues compared to normal tissues. Furthermore, there was a positive correlation between ETK expression and advanced clinicopathological features, while it was adversely linked with overall survival of patients. Subsequent investigations have shown that reducing the expression of ETK in renal cell carcinoma hinders the development and spread of cancer cells, and triggers programmed cell death, by reducing the levels of phosphorylated STAT3 (p-STAT3) and vascular endothelial growth factor (VEGF) [54]. In this study, it was demonstrated that ETK was a direct substrate protein of TRIM26, and was being ubiquitinated upon TRIM26 overexpression, which further led to the degradation of ETK. Furthermore, our findings demonstrated that the overexpression of ETK resulted in the upregulation of the AKT/mTOR signaling pathway in ccRCC, as shown by the elevated levels of phosphorylated AKT and phosphorylated mTOR. Rescue tests demonstrated that ETK mitigated the impact of TRIM26-induced deactivation of the AKT/mTOR signaling pathway. Compelling new data suggests that ETK triggers tyrosine phosphorylation of certain proteins, such as EGFR and STAT3, by direct binding [55]. Thus, it is plausible to hypothesize that the activation of AKT/mTOR signaling by ETK was achieved by its interaction with AKT or its upstream kinase, resulting in an elevation in their phosphorylation levels. Furthermore, in vitro assays revealed that an excessive amount of ETK might reduce the inhibitory effects of TRIM26 overexpression on malignant behaviors in ccRCC. In summary, it may be inferred that TRIM26 suppresses the proliferation, migration, and invasion of ccRCC cells by causing the degradation of ETK, resulting in the deactivation of AKT/mTOR signaling.

To summarize, our current investigation presents definitive proof that TRIM26 was significantly reduced in ccRCC, and that decreased expression of TRIM26 was associated with worse survival outcomes. TRIM26 expression level has promise as a novel biomarker for prognosticating the outcomes of ccRCC patients. TRIM26 directly interacted with ETK, leading to the promotion of ubiquitination and degradation of ETK. This, in turn, resulted in the deactivation of the AKT/mTOR signaling pathway, eventually leading to reduced proliferation, migration, and invasion of cancer cells. Therefore, it is of great interest to develop therapeutic approaches for ccRCC patients by restoring TRIM26 expression in tumor cells.

### Electronic supplementary material

Below is the link to the electronic supplementary material.

Supplementary Material 1

## Data Availability

Data related to the current study are available from the corresponding author on reasonable request.

## Abbreviations

TCGA: The Cancer Genome Atlas
GEO: Gene Expression Omnibus
ccRCC: Clear cell renal cell carcinoma
