# Supplementary material for: TRIM26 inhibits clear cell renal cell carcinoma progression through destabilizing ETK and thus inactivation of AKT/mTOR signaling
Source: J Transl Med. 2024 May 21;22:481. doi: 10.1186/s12967-024-05273-w (PMC11110379; doi:10.1186/s12967-024-05273-w)
Supplement: Supplementary file 1 — Supplementary Material 1 [file 12967_2024_5273_MOESM1_ESM.docx]

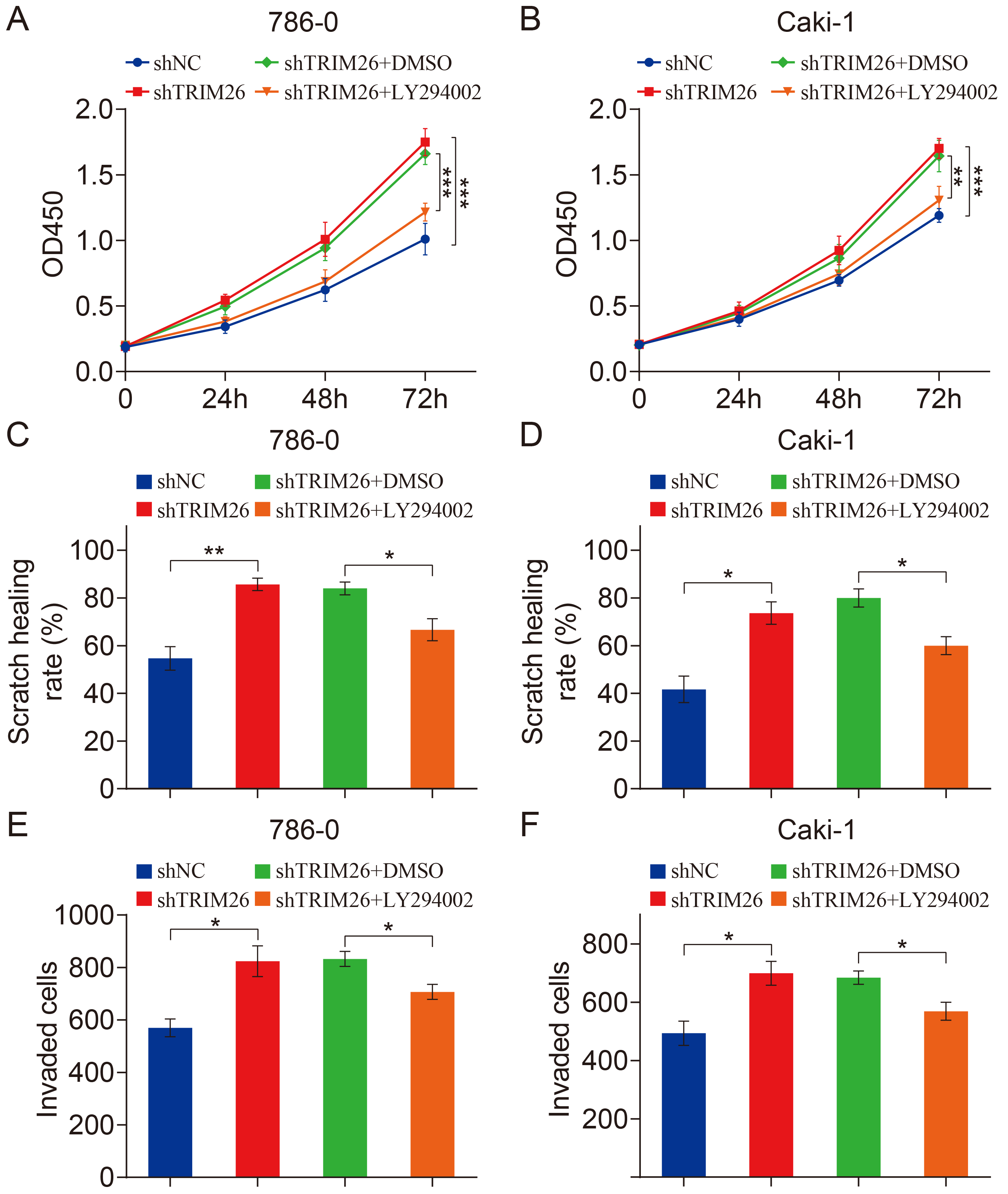


**Supplementary Figure 1. Silence of TRIM26 increased cell proliferation, migration, and invasion in ccRCC via activating AKT/mTOR signaling pathway.** (A-B) LY294002, an inhibitor of AKT/mTOR signaling, rescued TRIM26 silence induced increased proliferation ability. (C-D) Wound healing assay showing that cell migration was promoted after knocking down TRIM26, while this effect was reversed by the treatment of LY294002. (E-F) Transwell invasion assay revealed that LY294002 could partly attenuate TRIM26 silence induced increased invasion ability. **P*<0.05, ***P*<0.01, ****P*<0.001.


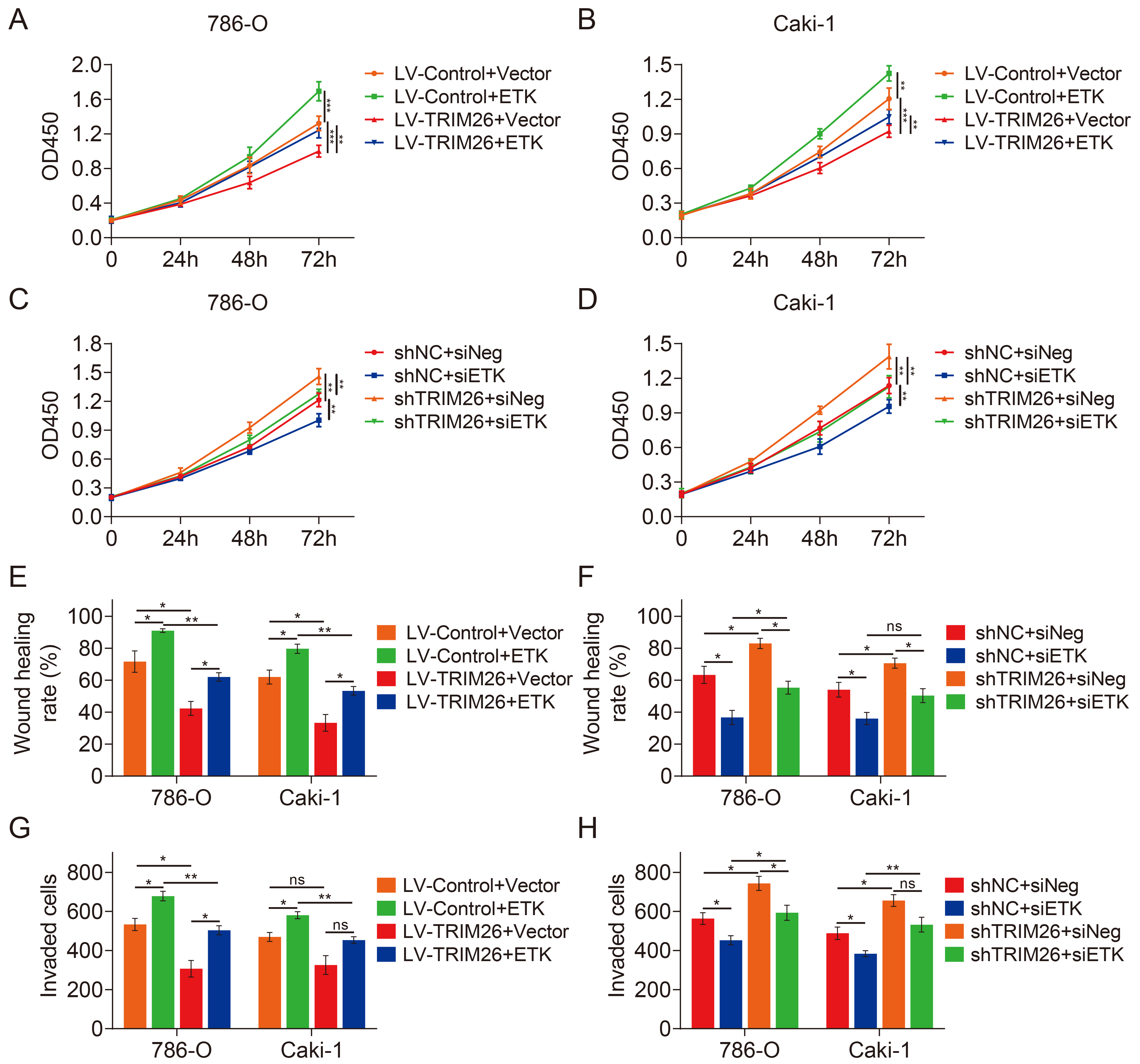


**Supplementary Figure 2. TRIM26 regulated cell proliferation, migration, and invasion via ETK.** (A-B) Cell proliferation abilities of 786-O and Caki-1 cells were impaired upon TRIM26 overexpression, which were further rescued by ETK overexpression. (C-D) Knocking down of ETK attenuated TRIM26 silence induced-increased cell proliferation. (E-F) Wound healing and transwell invasion assays revealed decreased cell migration and invasion abilities after overexpressing TRIM26, which were reversed by ETK overexpression. (G-H) Knocking down of ETK impaired TRIM26 silence induced-increased cell migration and invasion. **P*<0.05, ***P*<0.01, ****P*<0.001.
